# Identification of Regulatory Mutations in *SERPINC1* Affecting Vitamin D Response Elements Associated with Antithrombin Deficiency

**DOI:** 10.1371/journal.pone.0152159

**Published:** 2016-03-22

**Authors:** Mara Toderici, María Eugenia de la Morena-Barrio, José Padilla, Antonia Miñano, Ana Isabel Antón, Juan Antonio Iniesta, María Teresa Herranz, Nuria Fernández, Vicente Vicente, Javier Corral

**Affiliations:** 1 Servicio de Hematología y Oncología Médica, Hospital Universitario Morales Meseguer, Centro Regional de Hemodonación, Universidad de Murcia, IMIB-Arrixaca, Murcia, Spain; 2 Servicio de Neurología, Hospital Universitario Reina Sofía, Murcia, Spain; 3 Servicio de Medicina Interna, Hospital Universitario Morales Meseguer, Murcia, Spain; 4 Servicio de Hematología y Hemoterapia, Hospital Universitario Miguel Servet, Zaragoza, Spain; The University of Hong Kong, HONG KONG

## Abstract

Antithrombin is a crucial anticoagulant serpin whose even moderate deficiency significantly increases the risk of thrombosis. Most cases with antithrombin deficiency carried genetic defects affecting exons or flanking regions of *SERPINC1*.We aimed to identify regulatory mutations in*SERPINC1* through sequencing the promoter, intron 1 and 2 of this gene in 23 patients with antithrombin deficiency but without known genetic defects. Three cases with moderate antithrombin deficiency (63–78%) carried potential regulatory mutations. One located 200 bp before the initiation ATG and two in intron 1. These mutations disrupted two out of five potential vitamin D receptor elements (VDRE) identified in *SERPINC1* with different software. One genetic defect, c.42-1060_-1057dupTTGA, was a new low prevalent polymorphism (MAF: 0.01) with functional consequences on plasma antithrombin levels. The relevance of the vitamin D pathway on the regulation of *SERPINC1* was confirmed in a cell model. Incubation of HepG2 with paricalcitol, a vitamin D analog, increased dose-dependently the levels of *SERPINC1*transcripts and antithrombin released to the conditioned medium. This study shows further evidence of the transcriptional regulation of *SERPINC1* by vitamin D and first describes the functional and pathological relevance of mutations affecting VDRE of this gene. Our study opens new perspectives in the search of new genetic defects involved in antithrombin deficiency and the risk of thrombosis as well as in the design of new antithrombotic treatments.

## Introduction

Minor changes in the sequence of *SERPINC1*, the gene encoding antithrombin, have pathological consequences due to the functional and conformational sensitivity of this key anticoagulant [1]. Only few polymorphisms affecting *SERPINC1* are described, most of them neutral, and those with missense consequences, such as that responsible for the Cambridge II variant has been associated with increased risk of venous thrombosis [2].Thus, practically all mutations or gross deletions affecting the coding or flanking regions of *SERPINC1* cause antithrombin deficiency and are identified in 70–80% of patients with deficiency of this key anticoagulant [3,4]. These studies have also assisted to characterize functional or structural domains of antithrombin [5].

We speculated that the analysis of those cases with antithrombin deficiency that had no genetic defects in exons and flanking regions of *SERPINC1* might help to identify new mechanisms associated with deficiency of this key anticoagulant. Regulatory defects may potentially explain some of these unknown cases. Unfortunately, the information of regulatory aspects of *SERPINC1* is quite limited. The promoter lacks TATA box, and GC rich region [6–8]. Few studies, most of them using deletional analysis and reporter or DNase I footprint assays, have identified few regions potentially involved in the transcriptional regulation of this gene [8–11]. Sequencing of the 1500 bp of the potential promoter region in a family with moderate antithrombin deficiency allowed us to identify the first regulatory mutation (c.1-171 C>G) [12] affecting a region 200 bp upstream the ATG start codon with additional evidences supporting its regulatory relevance [9,13]. On the other hand, a relatively common polymorphism, rs2227589, located in intron 1 has been associated with a mild risk of venous thrombosis [14] and has mild functional consequences on the levels of antithrombin in plasma by an unknown mechanism [15].

The analysis of potential regulatory regions of *SERPINC1* in patients with antithrombin deficiency but without known genetic defects is an excellent strategy to identify new regulatory elements of *SERPINC1* and mechanisms involved in the regulation of antithrombin, and could potentially reveal new thrombotic risk factors.

## Material and Methods

### *In silico* search for vitamin D response elements (RXRα/VDR) in *SERPINC1*

The search for potential vitamin D response elements (VDRE) in *SERPINC1* was performed *in silico* by using three available software: JASPAR (http://jaspar.genereg.net/) [16] performing the 74.1 RXRA::VDR matrix and a threshold of 70 and 80% (S1 Fig); Genomatix (https://www.genomatix.de/); and PROMO (http://alggen.lsi.upc.es/cgi-bin/promo_v3/promo/promoinit.cgi?dirDB=TF_8.3) [17,18] using version 8.3 of TRANSFAC.

### Patients and healthy subjects

One hundred and fifty eight unrelated patients with confirmed reduced levels of antithrombin in plasma in at least two determinations(<80% of the reference plasma generated with plasma from 100 Caucasian blood donor, representative of the general population) were recruited during the last 10 years from different countries, mainly Spain. As the objective of our study was to find new mechanisms causing antithrombin deficiency, particularly gene variations with potential regulatory function of *SERPINC1*, inclusion criteria were patients with antithrombin deficiency but with no mutation or gross deletion/insertion in coding regions of *SERPINC1*. Thus, from the 158 patients with antithrombin deficiency recruited, 127 were excluded because they have mutation or gross deletion/insertion in the coding regions of *SERPINC1* able to explain the associated deficiency. Eight additional cases without mutations in *SERPINC1* were also excluded because they displayed an aberrant hypoglycosylation in plasma antithrombin and other N-glycoproteins, which explained the antithrombin deficiency (de la Morena-Barrio et al. unpublished results). Therefore, our study finally included 23 cases patients with antithrombin deficiency with unknown molecular defect.

As a control group we study a cohort of 307 Spanish Caucasian healthy blood donors (138 men/169 women) with an average age of 43 years.

### Blood sampling

Blood samples were obtained by venopuncture collection into 1:10 volume of trisodium citrate. Platelet poor plasma fractions were obtained (within 5 min after blood collection) by centrifugation at 4°C for 20 min at 2200*g* and stored at -70°C. Genomic DNA was purified by salting out procedure and stored at -20°C.

All subjects gave their written informed consent to enter the study, which was approved by the ethics committee of the Hospital Reina Sofia (number 8/2013) (Murcia, Spain) and performed according to the declaration of Helsinki, as amended in Edinburgh in 2000.

### Antithrombin levels

Plasma FXa-inhibiting activity was measured using a chromogenic method in presence of heparin (HemosIL TH, Instrumentation Laboratory) as previously reported [19]. Values were expressed as a percentage of the result observed in a pool of citrated plasma from 100 healthy subjects(mean: 97.1±6.6, 95% CI [95.8–98.4]).This reference plasmawas made pooling plasma from 100 healthy blood donor Caucasian. Blood was obtained by venopuncture collection into 1:10 volume of trisodium citrate. Platelet poor plasma fractions were obtained (within 5 min after blood collection) by centrifugation at 4°C for 20 min at 2200 *g*. All plasmas were pooled, aliquoted and stored at -70°C.

Antigen levels were determined by laurel immunoassay and ELISA.

Plasma antithrombin was also evaluated by electrophoretic analysis (using denaturing and native conditions with and without urea) and western blot following conditions and antibodies described elsewhere [20].

### Amplification and sequencing of *SERPINC1*

Polymerase chain reactions (PCR) covering the 7 exons and flanking regions as well as the potential promoter region, intron 1 and 2 of *SERPINC1*were carried out using Expand Long Template Polymerase (Roche, Spain) and the oligonucleotide sets described in S1 Table. PCR products were purified and sequenced with ABI Prism Big Dye Terminator v3.1 Cycle sequencing kit and resolved on a 3130xl Genetic Analyzer (Applied Biosystems, Spain). Comparison with the reference sequence (GenBank accession number NG_012462.1) was performed with SeqScape v2.5 software (Applied Biosystems, Spain).

### Multiplex Ligation-dependent Probe Amplification (MLPA)

Multiplex Ligation-dependent Probe Amplification technique, a multiplex PCR method able to distinguish sequences differing in only one nucleotide, was used to detect abnormal copy numbers of up to 50 different genomic DNA or RNA sequences. To specifically detect deletions or duplications in the *SERPINC1* gene we used the SALSA MLPA Kit P227 SerpinC1 (MRC-Holland); which uses probes that target all the 7 coding exons of *SERPINC1*.

### Genotyping of c.42-1060_-1057dupTTGAandc.42-1087_-1068dup by capillary electrophoresis

Intron 1 of *SERPINC1* was amplified by using the following oligonucleotides: F 5’GGCGACTATTAAAAATTCCAGGCA 3’ and R 5’ [FAM]CTCCTCCTCATTTAGTCAAACCCCA 3’. Amplified products were run in an automatic sequencer (3130 Applied Biosystem) and analyzed using the 3130 Applied Biosystem software.

Genotyping of these duplications was done in the group of healthy controls (N = 307) and in the group of patients with antithrombin deficiency carrying mutations in exons or flanking regions (N = 127).

Positive cases carrying duplications were validated by Sanger’s sequencing.

### Genotyping of *SERPINC1*rs146692719 and rs2227589 polymorphisms

Genotyping of the *SERPINC1*rs146692719polymorphism was done by PCR-ASRA with Tfi I, which is sensitive to this mutation, in 83 Spanish patients with venous thrombosis and 80healthy blood donor controls.

Genotyping of the *SERPINC1* rs2227589 polymorphism was done by the validated TaqMan SNP Genotyping Assay C__16180170_20 (Applied Biosystems) following the manufacturer’s instructions. Reactions were performed on a LC480 Real Time PCR (Roche) using standard conditions for end-point genotyping assays on this machine.

### Cell culture

HepG2 cell line was grown at 37°C, 5% CO2, in Dulbecco’s Modified Eagle Medium (DMEM, Invitrogen) supplemented with 10% foetal bovine serum (Sigma-Aldrich), penicillin (120 μg/ml) (Sigma-Aldrich), streptomycin (100μg/ml) (Sigma-Aldrich) and glucose (1 μg/ml) (Sigma-Aldrich). At 70% of confluence cells were seeded in a 24 well plate at 140000 cell/well of density in 0.5 ml/well of DMEM medium. After 24 hours, cells were washed with PBS. Then cells were grown for 24 hours in 300 μl of CD-CHO medium (Invitrogen) containing 0, 40, 80 and 106ng/μl of paricalcitol (Zemplar) (19-nor-1,25-(OH)2-vitamin D2, analogue of 1,25-dihydroxyergocalciferol, the active form of vitamin D2).

Antithrombin and prothrombin secreted to the conditioned medium was evaluated by electrophoretic analyses using SDS PAGE under reducing conditions as previously reported [20]. Samples were transblotted onto a PVDF membrane and immune stained with antibodies: rabbit anti-human antithrombin polyclonal antibody (A9522, Sigma-Adrich) and rabbit anti-human prothrombin polyclonal antibody (ab113431, Abcam), followed by donkey anti-rabbit IgG-horseradish peroxidase conjugate (NA9340V, GE Healthcare) with detection via an EC kit (Amersham Biosciences, Piscataway, NJ).

Cells were washed and lysed with a cell lysis buffer (RIPA lysis and extraction buffer, Thermo Fisher) containing protease and phosphatase inhibitor cocktails. RNA was purified using RNAzol RT (Molecular Research Center) following manufacturer instructions. *SERPINC1* and *VDR* gene expressions were determined by qRT-PCR by using specific TaqMan probes (Hs00166654m1 and Hs01045840m1, respectively) and β-actin constitutive reference gene (Hs99999903m1) (both from Applied Biosystem). The results were expressed as relative gene expression respect to the internal control gene β-actin using the comparative *C*_T_ method also referred to as the 2^-Δ^*^C^*^T^method.

### Statistical analyses

Association betweengenetic modifications and plasma anti-FXa activity was tested by a Student's *t*-test following a dominant model and adjusted for factors described to influence antithrombin levels (age, gender and the *SERPINC1* rs2227589 polymorphism, a functional polymorphism in intron 1) [15,21]. Data are presented as mean ± standard deviation. This analysis was carried out using the Statistical Package for Social Science (SPSS version 15.0, USA) considering statistically significant as p<0.05.

## Results

### *In silico* search for vitamin D response elements (RXRα/VDR) in *SERPINC1* gene

**Fig 1:**
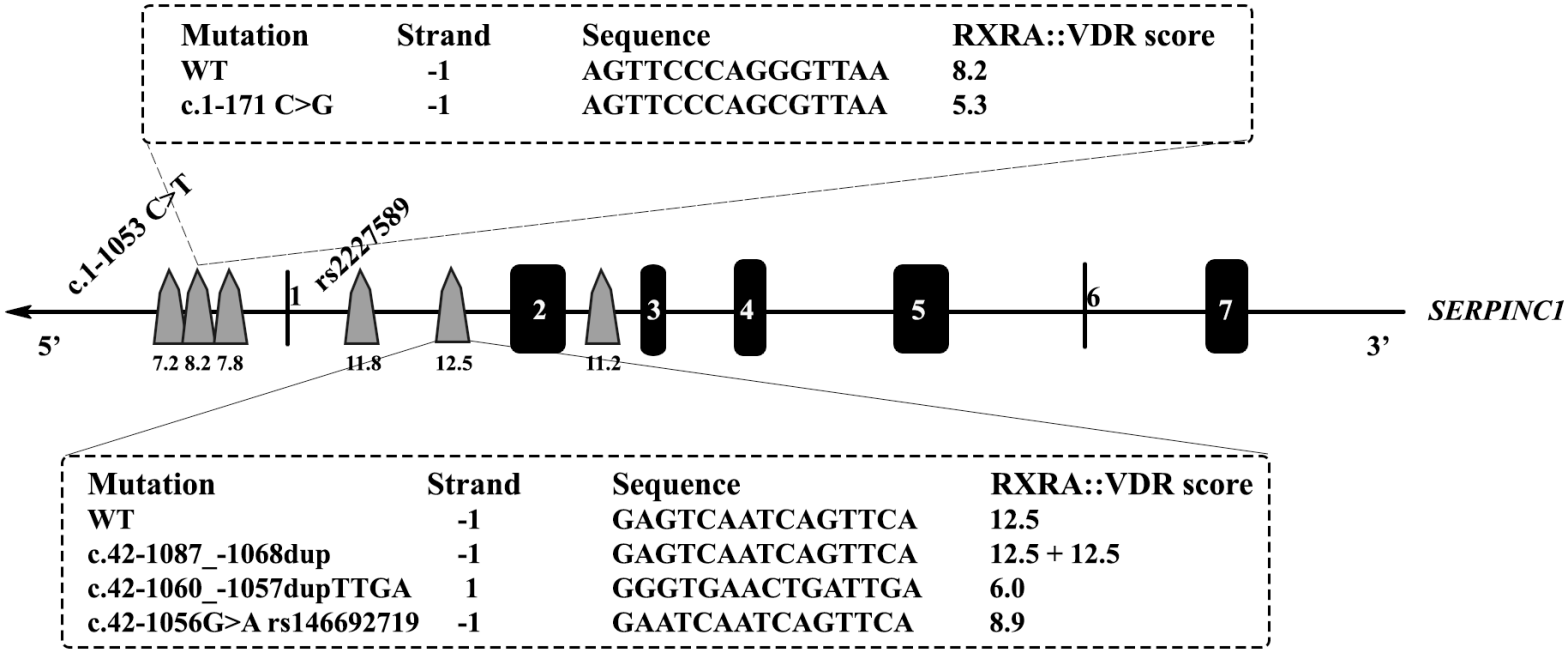
Localization in *SERPINC1* gene of potential vitamin D response elements (VDRE) identified by using *in silico* predictions with JASPAR software. The localization in *SERPINC1* of the genetic variations evaluated in this study is shown. The predictive score of interaction with RXRα/VDR of wild type and mutated sequences obtained by using JASPAR software are also indicated.

*In silico* prediction of potential regulatory regions affected by thec.1-171 C>G, described previously by our group in a patient with mild antithrombin deficiency [12] identified potential binding sites for hepatocyte nuclear factor alpha (HNFα) thyroid receptor alpha (TRα), chicken ovalbumin upstream promoter-transcription factor I (COUP-TFI), and retinoid X receptor alpha/vitamin D receptor (RXRα/VDR). Interestingly, JASPAR predicted two additional VDREs located very close(strand: -1 AAGTTTTAAAGTTCA; strand:-1, GGGGAACTGAGGTCA) all these three elements at 200 bp to the ATG initiation codon. These VDREs were confirmed by PROMO and Genomatix, in all predictions with high scores (Fig 1).This finding together with the evidences supporting a role of vitamin D in the control of *SERPINC1* [22] encouraged finding additional VDRE in *SERPINC1*. We screened the presence of additional VDREs in *SERPINC1* by JASPAR software. This analysis revealed 3potentially strong VDREs in *SERPINC1*; two in the intron 1 and one in theintron 2 with high predicted scores (11.2–12.5) (Fig 1). It is important to point out that the gene encoding parathyroid hormone (*PTH*), whose vitamin D transcriptional regulation is well known [23], has two VDRE in intron 1 predicted by JASPAR software with similar scores (11.5 and 14.8).

Finally, the functional polymorphism localized in intron 1, rs2227589 [14] did not affect any VDRE identified in this intron (Fig 1).

### Screening of new potential regulatory mutations in *SERPINC1*

Accordingly to the *in silico* results, we decided to sequence 1500 bp of the 5' region, intron 1 and intron 2 of *SERPINC1*, regions containing the potential VDRE regions, in 23 patients with antithrombin deficiency without mutations in coding exons and flanking regions or gross gene defects.

The new genetic variants identified in this study were submitted to the DNA databank of Japan (DDBJ) (accession numbers: LC127223, LC127224, LC127225, LC127226).

Two genetic modifications affecting the promoter region were identified in these 23 patients. The c.1-1053C>T mutation located at 1053bp from the ATG start codonwas identified in heterozygosis in a woman (P1) with mild antithrombin deficiency (73%) who suffered a cerebral thrombosis at the age of 27 years (Table 1). The c.1-1053 C>T point mutation that did not disturb any VDRE (Fig 1) was genotyped in 316 healthy blood donors. This study revealed that it is a new low prevalent polymorphism identified in heterozygosis in 5 out of 307 blood donors (MAF:0.008)that has no functional effect on the levels of antithrombin in plasma (anti-FXa activity: 96.3±7.5%in carriers *vs*97.5±6.6% in non carriers; p = 0.729).The low MAF of this polymorphism may require further studies, increasing the size of the sample, to fully discard any functional effect.

**Table 1:** Demographic, clinical, analytical and genetic features of patients with antithrombin deficiency and mutations affecting potential regulatory sequences in *SERPINC1*.

	Sex	Age (thrombosis)	Type of thrombosis	Risk factors*	Family history of thrombosis	Anti-FXa activity (%)	Genetic defect
**P1**	F	30 (27)	Cerebral thrombosis	No	No	73	c.1-1053 C>T
**P2**	F	64 (63)	PE	Obesity	No	69	c.1-171 C>G
**P3**	M	90 (88)	Cardioembolicstroke	AF, HT	No	68	c.42-1060_-1057dupTTGA
**P4**	F	44 (42)	PE & DVT	Cancer	No	78	c.42-1087_-1068dup

PE: pulmonary embolism; DVT: deep venous thrombosis.

*Thrombotic risk factors include: thrombophilia (FV Leiden, PT G20210A, protein S and protein C deficiency, and antiphospholipid antibodies) and acquired risk factor for thrombosis (smoking, obesity, hypertension–HT-, hypercholesterolemia, diabetes, atrial fibrillation–AF- or cancer).

Interestingly, the c.1-171 C>G mutation was identified in P2, a woman with mild antithrombin deficiency (69%) who developed an idiopathic bilateral pulmonary tromboembolism when she was 63 years old (Table 1). This case was unrelated to the first one carrying this mutation [12].

No genetic modification was detected by sequencing of PCR products of the *SERPINC1* intron 2 in the 23 selected patients.

In contrast, the analysis of intron 1 revealed two new interesting genetic variations in our cohort of patients.

One patient, P3, a man who suffered a cardioembolic stroke at the age of 88,had 68% of anti-FXa activity and carried in heterozygosis a duplication of 4 nucleotides (c.42-1060_-1057dupTTGA) (Table 1 and S2A Fig). This duplication disturbed the VDRE with the highest score located at intron 1 (Fig 1). The score of interaction with RXRα/VDR predicted by JASPAR was 52% reduced in the mutated allele compare to the score of the wild type sequence (Fig 1). Family studies performed in two sons with normal anti-FXa activity (100 and 90%) showed no carrier.

This duplication was genotyped in 307 healthy subjects by PCR and capillary electrophoresis. Positive cases were always confirmed by sequencing. The c.42-1060_-1057dupTTGA allele was found in 8 controls, always in heterozygous state, revealing that it is a new polymorphism with a low prevalence in our population (MAF: 0.014). Interestingly, this new polymorphism had functional effect on antithrombin levels in plasma, as carriers had lower plasma anti-FXa activity levels than non carriers (90.7±6.5% *vs*96.4±7.5%; respectively; p = 0.04). This difference maintained the statistical significance (p = 0.03) after adjustment by age, sex and by the *SERPINC1* functional polymorphism rs2227589, also located in intron 1.

Finally, this duplication was also genotyped in 127cases with antithrombin deficiency carrying a genetic defect in exons or flanking regions of *SERPINC1*. One case, a 32-year old woman with 52% anti-FXa who developed recurrent thrombophlebitis, carried this polymorphism in combination with a three base pair deletion in exon 5(CD930907) that maintaining the reading frame caused the deletion of a glutamic acid (p.Glu344del), mutation previously described by Chowdhury and coworkers in a patient with a type I deficiency [24].

The last potential regulatory genetic variation identified in patients with antithrombin deficiency but with not mutations or gross deletions in exons or flanking regions of *SERPINC1* was discovered in P4 (Table 1), an African woman with moderate antithrombin deficiency (78% of anti-FXa activity) who suffered a deep venous thrombosis and a pulmonary embolism at the age of 42,one month after receiving major chemotherapy due to an invasive ductal carcinoma. P4 carried a20bpduplication in intron 1 (c.42-1087_-1068dup)in heterozygosis (S2B Fig). JASPAR prediction revealed that the variant allele has duplicated the strongest RXRα/VDR element of intron 1 (Fig 1). This duplication was not found in 307 healthy controls. Unfortunately, we have not been able to study this mutation neither in African population nor in relatives of the proband. Further studies in subjects with the same genetic background are needed to define the prevalence, functional effect, and pathological consequences of this new alteration.

### Genotyping of rs146692719

The search of described genetic variations in *SERPINC1* affecting potential VDREs identified a polymorphism, rs146692719(c.42-1056G>A), which also disturbed the VDRE with highest score of intron 1 (Fig 1). The score of interaction with RXRα/VDR predicted by JASPAR was 29% reduced in the mutated allele (Fig 1). Accordingly to the low MAF of this variation (A: 0.0034/17 described in the 1000 genomes project) (http://www.ncbi.nlm.nih.gov/projects/SNP/snp_ref.cgi?rs=146692719) genotyping of this polymorphism in 83 Spanish patients with venous thrombosis and 80 controls revealed no carrier.

### *In vitro* effect of vitamin D on the transcriptional control of *SERPINC1* in HepG2 cell line

**Fig 2:**
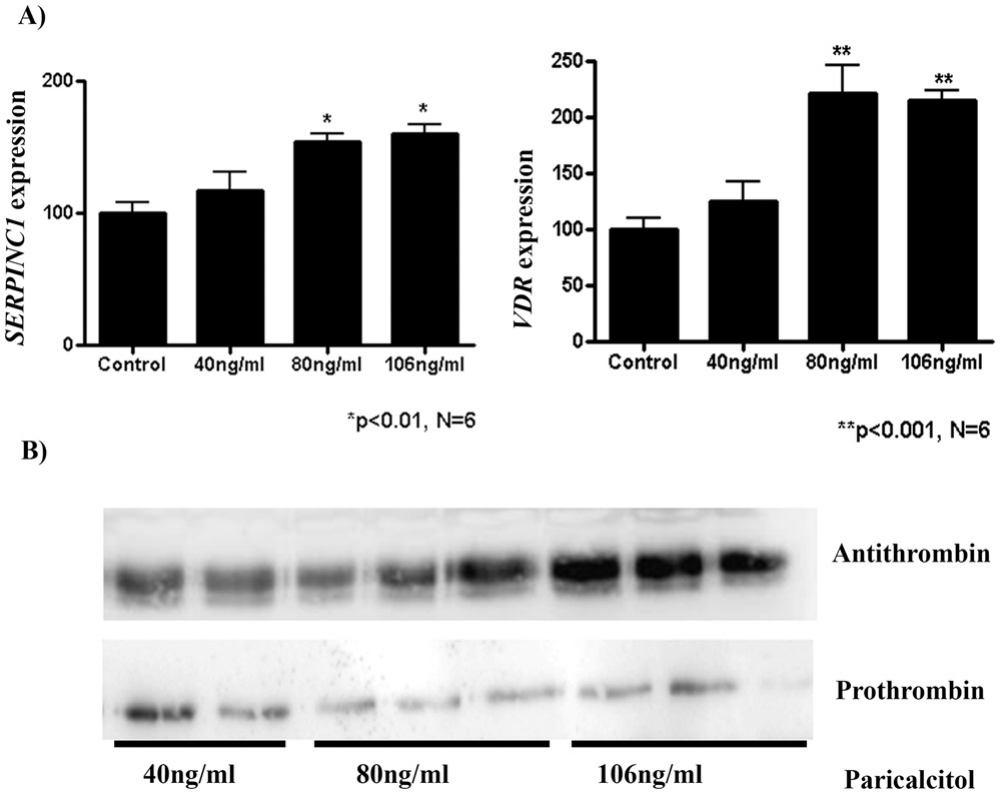
Effect of paricalcitol, a vitamin D analog, on HepG2 cells. A) RNA expression levels of *SERPINC1* and *VDR*. B) Western blot analysis of antithrombin and prothrombin secreted to the conditioned medium.

The treatment of HepG2 during 24 hours with different doses (40, 80 and 106ng/ml) of paricalcitol,a vitamin D analog, increased the levels of expression of *SERPINC1* mRNA in a dose dependent manner (p<0.01) (Fig 2A). The increased transcriptional rate paralleled with augmented levels of antithrombin secreted to the conditioned medium (Fig 2B). For the highest dose, a 1.5 fold increase of both transcription and antithrombin levels was observed. In contrast, paricalcitol had negligible effects on the levels of prothrombin in the conditioned medium (Fig 2B).

Moreover, mRNA expression of the vitamin D receptor gene (*VDR*) was also measured. The highest dose of paricalcitol significantly enhanced, up to 2 fold, the levels of *VDR* mRNA levels (p<0.001) (Fig 2A).

## Discussion

The key hemostatic role of antithrombin is sustained by the broad range of procoagulant proteases inhibited by this serpin and its efficient mechanism of inhibition [1]. Complete antithrombin deficiency caused embryonic lethality in mice [25] and triggers disseminated intravascular coagulation and thrombosis in Zebrafish [26]. Moreover, antithrombin deficiency was the first and so far strongest thrombophilic defect [27] and even minor reduction of antithrombin significantly increase the risk of thrombosis [28]. This relevance explains the high knowledge of this anticoagulant: the gene has been cloned and characterized in multiple species, up to 330different mutations involved in antithrombin deficiency have been identified, the crystal structure and the mechanism of activation and inhibition have been described [1]. However, it surprises the few information concerning regulatory mechanisms of such a crucial molecule. Two Genome Wide Association Studies (GWAS) aimed to identify modulating genes of antithrombin [29,30] with relatively poor results. Moreover, only few studies have searched for regulatory elements on *SERPINC1*. A small number of evidences support the regulatory relevance of a 200 bp region upstream the ATG start codon [8–11], but the regulatory pathway associated is unknown. We speculated that the sequence analysis of potential regulatory regions of *SERPINC1* in patients with antithrombin deficiency not explained by gene defects affecting exons and flanking regions might identify new regulatory elements and pathways.This strategy identified 3 gene defects disturbing two VDREs of *SERPINC1*, all with mild consequences on the levels of antithrombin in plasma. Two of these defects are restricted to the index family, but one, the c.42-1060_-1057dupTTGA affecting one VDRE of intron 1, is a new low prevalent polymorphism in Spain (MAF: 0.013) with moderate functional consequences. The functional consequences of the c.42-1060_-1057dupTTGA polymorphism might also modulate the risk of venous thrombosis in patients with other genetic defect causing antithrombin deficiency, as it has recently described for antithrombin Cambridge variants, a relatively frequent variation of *SERPINC1* [31].Finally, the potential role in thrombosis of this new polymorphism should be evaluated in large case/control studies.

Two studies suggested a potential role of vitamin D in the regulation of antithrombin levels. Vitamin D receptor knockout mice had a significant down regulation of the *serpinc1* expression in the liver and the levels of antithrombin in plasma in *vdr* -/- were 20% lower than in control mice [22]. Moreover, the combined treatment of HepG2 cells with 1,25-dihydroxy-cholecalciferol [1,25-(OH)2VitD3] and all-trans retinoic acid (ATRA), stimulated the production of antithrombin [32].The identification in this study of three genetic modifications disturbing two different VDRE sequences in patients with moderate antithrombin deficiency supports a role for this regulatory pathway in the *SERPINC1* transcriptional control. Finally, the dose-dependent response of *SERPINC1* mRNA and antithrombin levels to paricalcitol in HepG2 is new evidence supporting the role of vitamin D in the control of antithrombin.

The regulation of antithrombin by the vitamin D pathway might open new perspectives to explain the conflictive connection between vitamin D and thrombosis [33]. Multiple studies have provided indirect evidence for an inverse association between serum 25-hydroxyvitamin D (25(OH)D) and the predisposition to venous thromboembolism and other atherothrombotic diseases [34]. However, recent studies suggest that vitamin D deficiency does not play an important role in the pathogenesis of venous thrombosis [35,36]. Though, both the Tromsø and ARIC studies still maintain some doubts [35,36]. Additionally, supplementation with vitamin D seems to play a negligible effect, if any, on the risk of thrombosis [37], although some results, such as the reduced risk of idiopathic venous thromboembolisms in women randomized to calcium and vitamin D supplementation warrants further investigations [38]. Our study introduces new elements to play a role in this framework: genetic variations in VDRE of *SERPINC1*, which might exacerbate the consequences of a deficient status of vitamin D. It is possible that deficiency of vitamin D may only increase the risk of venous thrombosis in carriers of certain mutations or functional polymorphisms affecting elements involved in this pathway. Further studies, including analysis of the seasonal variation of antithrombin levels, as well as the effect of supplementation of vitamin D in carriers of VDRE mutations, are required to verify this hypothesis. Additionally, our study suggests that other elements of this pathway, such as the vitamin D receptor (*VDR*) or the retinoid X receptor (*RXR*), might also play a role in the variability of antithrombin in the general population, in antithrombin deficiency and in the risk of thrombosis. Further studies such as animal models of VDRE mutations supplemented with vitamin D will supply further evidences on the role of vitamin D and VDRE elements in the transcriptional control of *SERPINC1*.

Moreover, we also point out the potential therapeutic relevance of our results. Although very high levels of vitamin D, with no therapeutic application, are required to significantly increase the levels of antithrombin *in vivo*, it is possible that this supplementation therapy might be useful for some individuals with a genetic profile or in combination with other factors also regulating *SERPINC1*.

Finally, our results support the inclusion of regulatory regions in the molecular diagnosis of antithrombin deficiency, particularly in patients with moderate antithrombin deficiency. We propose that improved MLPA methods for diagnosis of antithrombin deficiency should also contain probes evaluating these regulatory regions.

## Supporting Information

S1 Fig Graphical representations of the matrix model used by JASPAR software to identify potential vitamin D response elements; sequences recognized by the RXRα/VDR complex. The information content of a matrix column ranges from 0 (no base preference) and 2 (only 1 base used). The sequence logo shows the total information content in each position, where the bar is replaced by stacked letters (A,C,G,T), which are sized and sorted relative to their occurrence.
(TIF)

S2 Fig Identification of genetic variants affecting the strongest VDRE of intron 1, c.42-1060_-1057dupTTGA (A) and c.42-1087_-1068dup (B) by capillary electrophoresis and sequencing. (TIF)

S1 Table Oligonucleotides used for PCR amplification of the *SERPINC1* gene. F: forward primer; R or B: reverse primer.
(DOCX)
