# Correction: Identification of Regulatory Mutations in *SERPINC1* Affecting Vitamin D Response Elements Associated with Antithrombin Deficiency

**DOI:** 10.1371/journal.pone.0159987

**Published:** 2016-07-21

**Authors:** Mara Toderici, María Eugenia de la Morena-Barrio, José Padilla, Antonia Miñano, Ana Isabel Antón, Juan Antonio Iniesta, María Teresa Herranz, Nuria Fernández, Vicente Vicente, Javier Corral

There are errors in the Funding section. The correct funding information is as follows: This work was supported by PI12/00657, PI15/00079; RD12/0042/0050, RD12/0042/0029, and CB15/00055 from ISCIII (Instituto de Salud Carlos III); Fundación Española de Trombosis y Hemostasia, and GATRA Grifols Award. MEM-B holds a post-doctoral contract from Ministerio de Economia y Competitividad FPDI-2013-17273.
