# Supplementary figures and images for: Identification of Regulatory Mutations in SERPINC1 Affecting Vitamin D Response Elements Associated with Antithrombin Deficiency
Source: PLoS One. 2016 Mar 22;11(3):e0152159. doi: 10.1371/journal.pone.0152159 (PMC4803246; doi:10.1371/journal.pone.0152159)

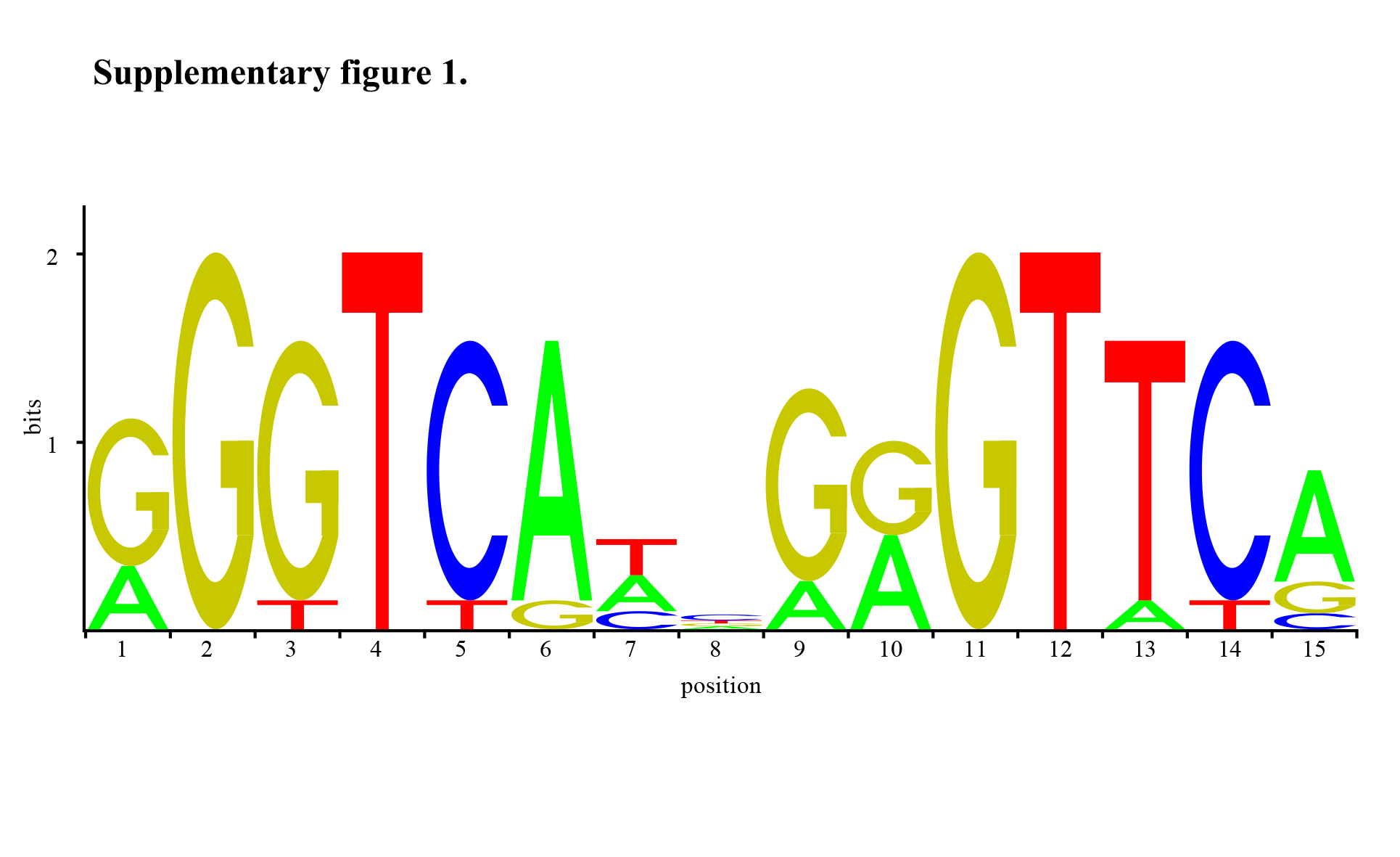

Supplement: S1 Fig — The information content of a matrix column ranges from 0 (no base preference) and 2 (only 1 base used). The sequence logo shows the total information content in each position, where the bar is replaced by stacked letters (A,C,G,T), which are sized and sorted relative to their occurrence. (TIF) [file pone.0152159.s001.tif]

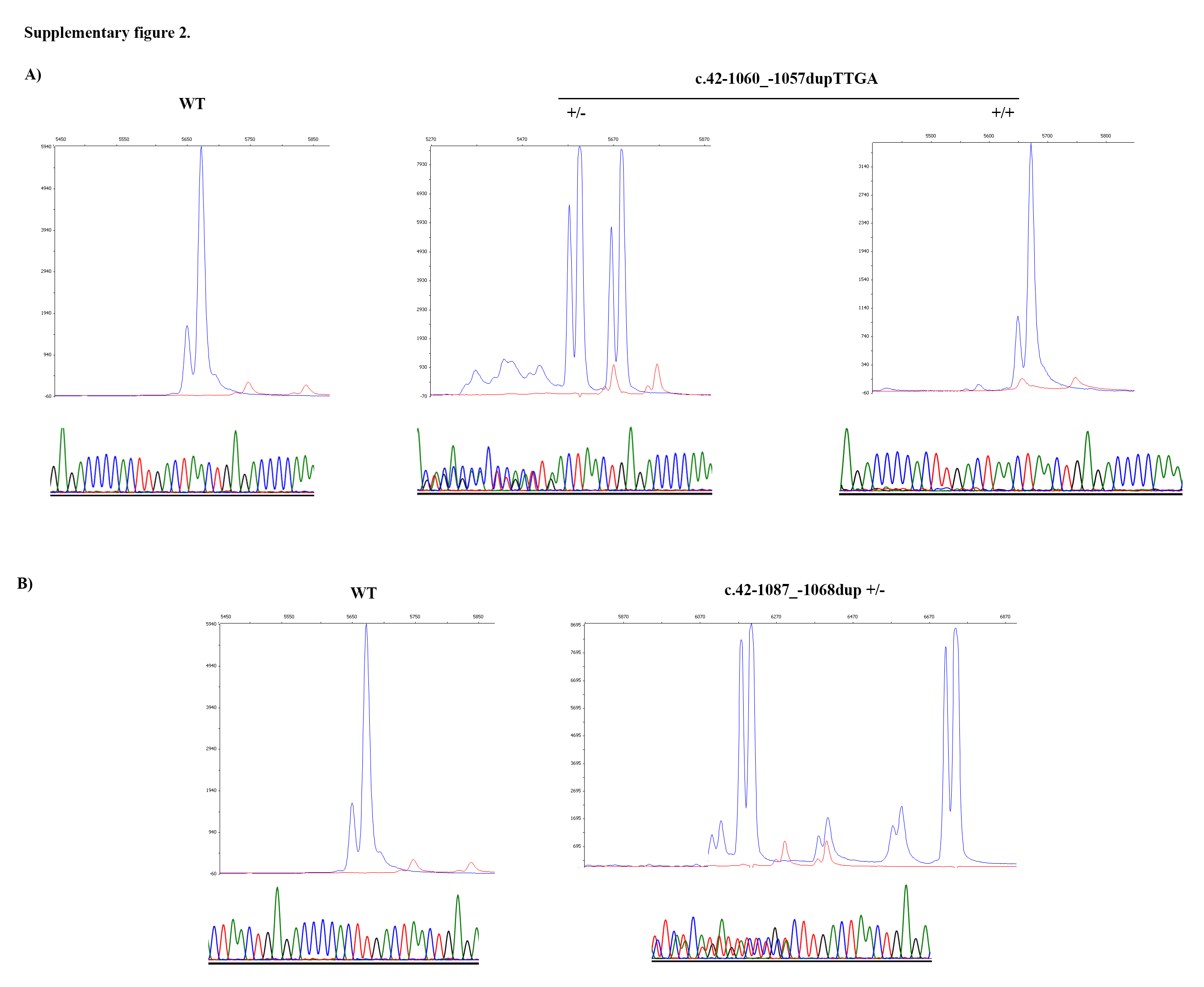

Supplement: S2 Fig — (TIF) [file pone.0152159.s002.tif]
