# Supplementary material for: Identification of Regulatory Mutations in SERPINC1 Affecting Vitamin D Response Elements Associated with Antithrombin Deficiency
Source: PLoS One. 2016 Mar 22;11(3):e0152159. doi: 10.1371/journal.pone.0152159 (PMC4803246; doi:10.1371/journal.pone.0152159)
Supplement: S1 Table — F: forward primer; R or B: reverse primer. (DOCX) [file pone.0152159.s003.docx]

**Supporting Information**

**S1 Table. Oligonucleotides used for PCR amplification of the *SERPINC1* gene.** F: forward primer; R or B: reverse primer.

| **Amplicon** | **Primer name** | **Oligonucleotide sequence (5´-3’)** | **Size (bp)** |
| --- | --- | --- | --- |
| **5’ region** | AT_-1F | GGACCTTATTAACATCTGAC | 743 |
|  | AT_-728R | CCAAGTTCACAGGGCGCATG |  |
|  | AT_-660F | GGAGTCCTTGATCACACAGCA | 1088 |
|  | AT_IN1R | GTCTTTGACTGTAACTACCAG |  |
| **Exon 1** | AT1F | CTCTGGAACCTCTGCGAGA | 197 |
|  | AT1B | GAAAGCTCACCCCTCTTAC |  |
| **Exon 2** | AT2F | TGCAGCCTAGCTTAACTTGGCA | 500 |
|  | AT2B2 | GGTTGAGGAATCATTGGACTTG |  |
| **Exon 3** | ATex3F | TGTGCTCACCACCCATGTTA | 320 |
|  | ATex3R | ATGCTGTTTCTCCACCTCCT |  |
| **Exon 4** | ATex4F | AAGCCAATTGAATAGCACAGG | 210 |
|  | ATex4R | AAGGGGGTAAGCTGAAGAG |  |
| **Exon 5** | AT5F | TGTGTTCTTACTTTGTGATTCTCT | 402 |
|  | AT5B | AAGGGAGGAAACTCCTTCCTAG |  |
| **Exon 6** | AT6F | TTCTCCCATCTCACAAAGAC | 232 |
|  | AT115R | CCACAGGCCTGCTATAATACAG |  |
| **Exon 7** | ATex7F | CTGTGGATGATTTACCTGCC | 351 |
|  | ATex7R | GCCCCAATAGCATGTTTCCCC |  |

**S1 Fig**. Graphical representations of the matrix model used by JASPAR software to identify potential vitamin D response elements; sequences recognized by the RXRα/VDR complex. The information content of a matrix column ranges from 0 (no base preference) and 2 (only 1 base used). The sequence logo shows the total information content in each position, where the bar is replaced by stacked letters (A,C,G,T), which are sized and sorted relative to their occurrence.

**S2 Fig.** Identification of genetic variants affecting the strongest VDRE of intron 1, c.42-1060_-1057dupTTGA (A) and c.42-1087_-1068dup (B) by capillary electrophoresis and sequencing.
